# Sarcopenic Obesity in Heart Failure With Preserved Ejection Fraction

**DOI:** 10.3389/fendo.2020.558271

**Published:** 2020-09-30

**Authors:** Danielle L. Kirkman, Natalie Bohmke, Hayley E. Billingsley, Salvatore Carbone

**Affiliations:** Department of Kinesiology and Health Sciences, College of Humanities & Sciences, Virginia Commonwealth University, Richmond, VA, United States

**Keywords:** sarcopenia, obesity, heart failure, diastolic, exercise tolerance, quality of life, nutrition, exercise training

## Abstract

Heart failure with preserved ejection fraction (HFpEF) is a public health epidemic that is projected to double over the next two decades. Despite the high prevalence of HFpEF, there are currently no FDA approved therapies for health-related outcomes in this clinical syndrome making it one the greatest unmet needs in cardiovascular medicine. Aging and obesity are hallmarks of HFpEF and therefore there is a high incidence of sarcopenic obesity (SO) associated with this syndrome. The presence of SO in HFpEF patients is noteworthy as it is associated with co-morbidities, worsened cardiovascular health, hospitalizations, quality of life, and mortality. Furthermore, SO plays a central role in exercise intolerance, the most commonly reported clinical symptom of this condition. The aim of this review is to provide insights into the current knowledge pertaining to the contributing pathophysiological mechanisms and clinical outcomes associated with HFpEF-related SO. Current and prospective therapies to address SO in HFpEF, including lifestyle and pharmaceutical approaches, are discussed. The urgent need for future research aimed at better understanding the multifaceted physiological contributions to SO in HFpEF and implementing interventional strategies to specifically target SO is highlighted.

## Introduction

Heart failure (HF) is a rapidly growing public health epidemic affecting over 6.2 million Americans, with about half having HF with preserved ejection fraction (HFpEF) (1). As the American population continues to age, an increase of 66% in the age group over 80 years old is predicted by 2030 (2). In addition, the obesity epidemic in the US continues to grow, with 1 in 2 adults projected to be obese by 2030 (3). As a result of the growing aging and obese population, the prevalence of HF is expected to increase across all sex, racial, and ethnic groups (2). HF prevalence is projected to increase 46% by 2030 to affect over 8 million US adults (2). Importantly, despite the high prevalence of HFpEF, there are currently no FDA approved therapies for health-related outcomes in this clinical syndrome. Thus, HFpEF treatment is one of the greatest unmet needs in cardiovascular medicine.

The diagnosis of HFpEF remains a clinical challenge in cardiology, however, HFpEF is typically characterized by an ejection fraction >50% accompanied by diastolic dysfunction, high filling pressures and exercise intolerance (4). The complexity of its diagnosis and the recent proposed algorithms to identify patients with HFpEF have been discussed elsewhere (5).

Within this clinical syndrome, there are distinct phenotypes emerging that could potentially redefine how we design clinical trials and treat HFpEF patients (6, 7). The identification of the phenotypes of HFpEF would potentially allow to define the most appropriate and tailored therapeutic strategy. This approach has been proposed to finally allow to identify an effective therapy in patients with HFpEF that could be applied in clinical practice.

In regard to the identification of HFpEF phenotypes, obesity has gained attention as a potential one of HFpEF (8, 9), with targeted treatment of obesity hypothesized to improve syndrome-specific health-related outcomes (10). Indeed, HFpEF is now the most common HF associated with obesity (11, 12). Specifically, therapies that have been in the past utilized in patients with obesity, but without HFpEF, such as exercise training and caloric restriction-induced weight loss, have recently proven beneficial also in patients with obesity and concomitant HFpEF, at least with regards to exercise capacity and patient reported quality of life (QoL). Long-term effects of such strategies are clearly required to confirm these initial promising findings, as discussed at length in the next paragraphs.

Toward developing effective therapies for these patients, patient reported QoL has emerged as a predominant outcome measure, with an understanding of the physiological contributors to QoL emerging as an important area of investigation. Exercise intolerance is the most commonly reported symptom of HFpEF. The downstream consequences of exercise intolerance include reduced physical activity levels and physical function that have detrimental implications on patient QoL, especially in those individuals with HFpEF who are younger and with concomitant obesity and diabetes mellitus (13). While the manifestations of HFpEF-related exercise intolerance are multifaceted, there has recently been a paradigm shift away from the heart and toward the periphery as the major limitation of exercise capacity. In this respect, abnormalities in body composition may play an important role in exercise intolerance and may be an attractive therapeutic target to improve QoL in these patients. Of note, therapies such as exercise training that have targeted the extra-cardiac peripheral abnormalities (i.e., body composition), have shown beneficial effects in patients with HFpEF, despite little effect on cardiac function (14–17). These results propose that targeting non-cardiac abnormalities could potentially result in improved clinical outcomes in this population, although therapies that may also improve cardiac dysfunction would be desirable.

Sarcopenic obesity (SO), a clinical and functional condition defined by the coexistence of excess adiposity (i.e., obesity) with a decline in muscle mass and related strength and functionality (i.e., sarcopenia), is consistently reported in HFpEF patients and poses as a major limitation to exercise capacity (14). Patients with SO present with reduced exercise capacity characteristic of obesity (18), but also those associated with sarcopenia in the setting of HFpEF (19).

The aim of this review is to provide insights into the contributing physiological mechanisms and outcomes associated with HFpEF-related SO toward improving exercise intolerance and potentially QoL.

## Sarcopenic Obesity: Definition and Diagnosis

SO is commonly defined as the coexistence of sarcopenia and obesity. A recent systematic review of the definitions and diagnosis of sarcopenic obesity reported large heterogeneity in the definition of SO and diagnostic approaches (20). This is likely due to differences in the definitions of sarcopenia and obesity as well as the large range of methodologies employed to assess body composition and strength (20). Despite these limitations, SO is typically diagnosed by the presence of sarcopenia and obesity, each evaluated as separate parameters (21). Future directions in the field are aimed at determining whether SO should be diagnosed based on one composite parameter (20). This is based on the thinking that a synergistic relationship between reduced muscle mass and increased adiposity may result in an independent clinical phenotype (20). A composite diagnostic criterion in this case would have to employ concomitant evaluations of fat mass and fat-free mass (20). As this measure is yet to be determined and established, the separate definitions and diagnoses of sarcopenia and obesity are provided herein.

### Sarcopenia

A recently revised consensus from the European Working Group on Sarcopenia in Older People (EWGSOP) defined sarcopenia as a progressive skeletal muscle disorder that increases the risk of adverse physical outcomes such as falls, fractures, impaired physical function, disability, and mortality (21). While former sarcopenia diagnoses were predominantly based on the reduction of lean mass, the recent consensus prioritizes the presence of reduced muscle strength (dynapenia) and/or physical function in the diagnosis of sarcopenia due to its superiority in predicting adverse events (21). In addition to reductions in functional measures, concurrent declines in muscle mass (myopenia) (22) are also required for a sarcopenia diagnosis (Table 1) (21). With regards to measurements of strength in the diagnosis of sarcopenia, handgrip strength (21), or knee extensor strength (20) by dynamometry are recommended. Alternatively, the 30 s sit to stand test provides a measure of lower body strength (21). Physical function can be assessed by gait speed, the timed up and go (a measure of speed and agility), the 400 m walk test or the short physical performance battery (SPPB; gait, balance, and lower body strength) (21). Recommended methods of skeletal muscle mass measurement are bioelectrical impedance analysis (BIA), dual energy X-ray absorptiometry (DXA) or the gold standard magnetic resonance imaging (MRI) (20, 21). If these techniques are unavailable, although not optimal, calf circumference can be utilized (21). Readers are referred to the EWGSOP consensus for the sarcopenia diagnostic cut off criteria for the abovementioned methodologies (21).

**Table 1:** Diagnostic criteria for sarcopenia.

	**Dynapenia**	**Myopenia**	**Probable Sarcopenia**	**Confirmed sarcopenia**	**Confirmed sarcopenia**	**Severe sarcopenia**
Low muscle strength	✓		✓	✓	✓	✓
Low muscle quantity		✓		✓		✓
Low physical performance					✓	✓

*Adapted from (15, 16, 19)*.

### Obesity

Obesity is defined as an excessive accumulation and storage of fat in the body that impairs health. Commonly used methodology to diagnose obesity in SO are body mass index (BMI), waist circumference (WC), and when BIA or DXA are available, with total body fat mass percentage (20). There has been limited research into which is the most effective measure of obesity in predicting outcomes in the setting of SO. However, a recent study by Khor et al. (23) comparing the use of BMI, WC and fat mass percentage by DXA in the diagnosis of SO revealed that the different methodologies markedly increased the variation in SO prevalence. In this respect, SO prevalence was lowest when BMI was used in comparison to WC and fat mass percentage (23). The findings also showed that WC was most strongly correlated with SO related impairments in physical function (23), supporting previous findings that regional adipose tissue distribution is important in determining functional impairments in SO (24). In this respect, in addition to abdominal adiposity, increased intermuscular fat deposition may be a pathophysiological contributor to dynapenia and impaired physical function that is unique to SO (24, 25).

In the recent years, the role of obesity on atrial function, in addition to ventricles and skeletal muscle has received much attention (4). Of note, left atrial has shown to provide important prognostic information in patients with HFpEF, independent on the presence of obesity; however, when obesity is present, there is a significant increase in right atrial pressure with exercise, specifically, an increased right atrial pressure/peak oxygen consumption has been shown. This suggest that to achieve a given peak oxygen consumption, patients with obesity, and HFpEF reach significantly greater right atrial pressure. This highlights the important role of the left atrial in determining exercise capacity in patients with HFpEF, in which atrial myopathy is often found, and proposed to be a major substrate for the development of atrial fibrillation, which is, in fact, one of the most common comorbidities reported in this population. Interestingly, patients with obesity and HFpEF present an increased epicardial adipose tissue (EAT). The thickness of EAT is significantly associated with greater body mass index (BMI) and right atrial pressure, and is inversely associated with reduced cardiorespiratory fitness (5–8), except for one study, where the association was paradoxically positive, suggesting a beneficial effects of EAT (9). Although the direct effects of EAT are not entirely clear, it has been speculated that EAT may cause a pericardial restraint, potentially exacerbated by mediastinal fat or chest wall issues. In addition, the detrimental indirect effects of EAT that have been proposed are numerous; however, the proinflammatory effects of EAT have been proposed to drive most of the inflammation found in the myocardium itself, as opposed to the non-EAT, which may drive the low-grade systemic inflammation characteristic of obesity and HFpEF (10).

## Sarcopenic Obesity in HFpEF

Both sarcopenia and obesity are well-documented as separate entities in HFpEF patients (25, 26). Firstly, aging represents the largest risk factor for cardiovascular disease and HFpEF is the predominant form of HF in the aged population (27). The development of sarcopenia and increased adiposity are elemental sequalae of the aging process (28) and therefore elderly HFpEF patients are already at risk of SO as a function of age. In addition, HFpEF is typically accompanied by a plethora of co-morbidities such as type 2 diabetes mellitus, chronic obstructive pulmonary disease (COPD) and chronic kidney disease (CKD), the pathophysiology of which contribute to “accelerated aging.” This not only augments SO in the elderly HFpEF cohort, but it also prematurely predisposes younger individuals with HFpEF to aging related SO phenomena. Secondly, overweight and obesity are highly prevalent in HFpEF affecting >80% of patients (9, 29, 30). In this respect, obesity is emerging as a distinct phenotype of HFpEF (8, 9, 13, 31). Despite a lack of studies in the HFpEF population reporting SO as a single entity, the high incidence of sarcopenia and obesity as separate parameters is indicative of a widespread SO prevalence in these patients.

## Clinical Consequences of Sarcopenic Obesity in HFpEF

### Hospitalizations and Survival

Obesity is a clear risk factor for HF, particularly HFpEF (32). Such effects have been recently proposed to be mediated by the reduced cardiorespiratory fitness (CRF) characteristic of obesity (33). Once HF is diagnosed, however, the effects of increased BMI on clinical outcomes is quite complex, with overweight and obesity being associated with improved clinical outcomes in both HFrEF and HFpEF, at least in observational studies (34). A sub-analysis of the I-PRESERVED trial investigated the association of BMI and adverse outcomes in 4,019 HFpEF patients (29). These analyses showed a U-shaped relationship between BMI and the primary composite endpoint of all-cause mortality and HF-related hospitalizations (29). The adjusted risk of for all-cause mortality and HF-related hospitalizations was significantly greater in patients with a BMI ≥35 kg/m^2^ and patients with a BMI <23.5 kg/m^2^ compared with BMI categories 23.5–26.4 kg/m^2^, 26.5–30.9 kg/m^2^, and 31–34.9 kg/m^2^ (29). A similar analysis of the CHARM study that included 7,599 HF patients, showed similar findings in the cohort of HF patients with preserved ejection fraction (ejection fraction >40%) (30). The difference between these studies was that in the I-PRESERVED trial, patients with a BMI between 16.5 and 30.9 kg/m^2^ had the lowest risk of mortality and HF related hospitalization (29). In comparison, in the CHARM trial, the risk was lowest in obese patients with a BMI between 30 and 34.9 kg/m^2^ (30). This finding has been replicated in other large epidemiological studies of HF, introducing an “obesity paradox” whereby obesity appears to have a protective effect in HF (35–37). Whether the obesity paradox exists in the HFpEF cohort of HF is keenly debated and warrants further investigation (9, 35, 38). Nonetheless, a consistent finding is that underweight patients with HFpEF, and those who lose weight over time in the absence of a structured lifestyle intervention have an increased risk of all-cause mortality and HF hospitalization rates (29, 30, 39). This demonstrates the detrimental effects of unintentional weight loss, likely resulting in reduced lean mass, on clinical outcome in these patients. Toward gaining a clearer understanding of the obesity paradox, future studies in HFpEF that investigate the effects of concomitant sarcopenia and obesity on clinical outcomes are urgently needed. In such studies, the inclusion of CRF, muscle strength and impaired physical function alongside body composition measures would provide a more holistic insight toward identifying specific treatment targets to improve clinical outcomes in this complex syndrome (35, 38).

### Cardiovascular Outcomes

Whether obesity is a driver of cardiac dysfunction in HFpEF or whether it coexists alongside HFpEF, influencing the presentation of the syndrome remains controversial (8, 10, 40). A study by Obakata et al. compared HFpEF patients with obesity with HFpEF patients without obesity and non-obese healthy controls. Their findings showed that HFpEF patients with obesity had significantly increased estimated plasma volume, abnormal right, and left heart cardiac structure and increased ventricular filling pressures during exercise (8). This data points toward a central role of obesity in the development and progression of HFpEF (8). The study, however, did not include a group of obese individuals without HF, making it impossible to differentiate whether the effects reported were specifically resulting from the HFpEF obesity phenotype, or whether individuals with a obesity would have reported similar findings (10). Alternatively, Carbone et al. reported no relationship between obesity and measures of systolic and diastolic cardiac function assessed by echocardiography at rest and at peak exercise (18). Additionally, although weight loss following caloric restriction in HFpEF patients and concomitant obesity, despite improved CRF, it has no clinically significant effects on diastolic function (14).

There is currently little directly linking sarcopenia to cardiac abnormalities in HFpEF. It is speculated that inflammation and endocrine changes related to myopenia, in addition to a sedentary lifestyle, secondary to sarcopenia, provide a pathophysiological environment conducive to cardiac diastolic dysfunction (41, 42). Likewise, the role of SO in driving cardiac abnormalities in HFpEF remains undetermined. In the SICA-HF study of 117 HF patients, the quartile of patients with the worst diastolic function and estimated filling pressures (E/e′ >15) reported the lowest levels of appendicular lean mass (i.e., lean mass of extremities) and muscle strength (43). A recent retrospective analysis of 733 Koreans investigated the role of SO on cardiac parameters in individuals without overt cardiovascular disease (42). The analyses compared individuals with obesity and without obesity, with and without concurrent sarcopenia (42). When controlling for confounding variables, those with SO had the highest risk for diastolic dysfunction compared to those that had only sarcopenia or obesity (42). This suggests a potential role of SO in cardiac dysfunction which appears to be driven by synergistic crosstalk between reduced lean mass and excess adiposity, however, these data need to be followed up in studies of HFpEF patients.

### Quality of Life

Due to the discouraging lack of positive clinical trials to improve outcome in HFpEF, there has been a shift toward strategies that focus on improving patient QoL as the primary endpoint. A recent amalgamation of leading HFpEF clinical trial data from NEAT-HFpEF, INDIE-HFpEF, and RELAX by Reddy et al. assessed relationships between HFpEF severity variables and QoL (13). With participants split into tertiles based upon QoL, the HFpEF group with worst QoL had the highest BMI and the greatest prevalence of class 2 obesity (13). After adjusting for age, sex, and BMI, QoL was worst in those with the lowest physical activity and physical function measures (11). Furthermore, the SICA-HF study demonstrates a positive correlation between appendicular lean mass, muscle strength and QoL (43). These data hold promise for SO as a potential therapeutic target to improve patient reported QoL.

### Exercise Intolerance

As mentioned previously, exercise tolerance is the most common reported symptom of HFpEF and holds promise as a therapeutic target to improve patient reported QoL in this patient population (44). In these patients, obesity is consistently linked with a reduction in cardiorespiratory fitness, assessed as maximal oxygen uptake (VO_2_peak), the gold standard measurement of exercise capacity (8, 31). In this respect, the regional distribution of adipose tissue plays an integral role in HFpEF related exercise intolerance (24). Intra-Abdominal fat is the strongest independent predictor of VO_2_peak, with abdominal subcutaneous fat, thigh subcutaneous fat and thigh intramuscular fat: skeletal muscle ratio all negatively associated with VO_2_peak (24). The underlying mechanisms of obesity-related reduction in exercise capacity in HFpEF are not yet fully elucidated. As diastolic dysfunction has previously been reported as a central limitation to exercise capacity (45), it is reasonable to infer that the obesity driven changes in cardiac function may partly explain these reductions. However, a paradigm shift toward the periphery implicates skeletal muscle abnormalities that are synonymous with sarcopenia as the predominant limitation of exercise capacity in these patients (46). In this regard, aberrant skeletal muscle structure and function reported in HFpEF patients hamper oxygen extraction and utilization during exercise with deleterious consequences on exercise capacity (46, 47). Readers are referred to in-depth reviews detailing skeletal muscle dysfunctions and their role in exercise intolerance in HF (25, 48). In support of this paradigm, appendicular muscle mass, and skeletal muscle mass are strongly associated with VO_2_peak and physical function in HFpEF, even when adjusting for other contributing factors to exercise intolerance such as iron deficiency, pulmonary function, and New York Heart Association (NYHA) class (43). The effect of coexisting sarcopenia and obesity on HFpEF related exercise intolerance is yet to be delineated. However, an elegant study by Zamani et al. has recently demonstrated that adiposity is inversely associated with local skeletal muscle oxygen consumption in HFpEF patients (47). In addition, in the previously mentioned retrospective analysis of “healthy” Koreans without overt cardiovascular disease, individuals with SO reported significantly lower VO_2_peak compared with those who only had sarcopenia or obesity (42). This supports the idea of synergistic cross-talk between adipose tissue and skeletal muscle that is deleterious to functional capacity. Future investigations into the effects of adipose tissue on skeletal muscle in the setting of HFpEF are warranted.

In addition, patients with obesity and HFpEF present with greater plasma volume and increased filling pressures, which have not been consistently identified in patients without HFpEF (1). Moreover, patients with obesity present a greater septal flattening, likely resulting from a greater external constraints on the heart caused by obesity (1). In addition, when obesity and HFpEF coexist, despite similar metabolic work, the measured external work reported is significantly lower (1). Finally, patients with the obesity/HFpEF phenotype seem to respond less to decongestive therapies (2).

## Pathophysiology of Sarcopenic Obesity in HFpEF

**Figure 1:**
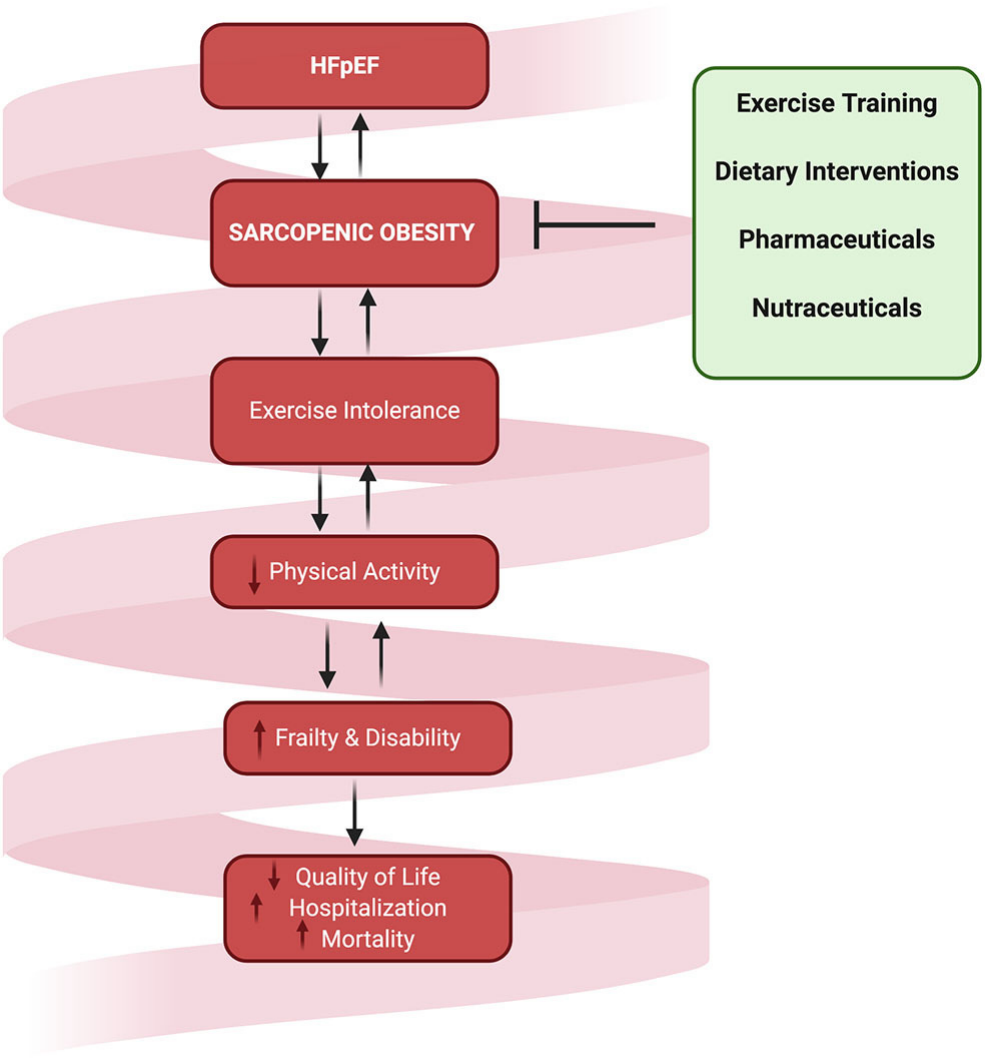
The multifaced pathophysiological contributors to sarcopenia in HFpEF. These factors all contribute to physical inactivity which results in reduced energy expenditure and disuse atrophy, initiating a deleterious vicious cycle between physical inactivity, co-morbidity, and augmented SO.

An overview of the multifaceted pathophysiological mechanisms that contribute to HFpEF related SO are highlighted in Figure 1.

### Inflammation

There is abundant evidence for inflammation in patients with HFpEF with circulating biomarkers of inflammation consistently reported to be substantially high in these patients (49, 50). Systemic inflammation is high in these patients due to volume overload that increases levels of angiotensin II, triggering a subsequent immune cascade; systemic microvascular inflammation; and the presence of co-morbidities such as hypertension, diabetes, anemia, chronic kidney disease, ischemic heart disease, stroke, and cancer are all marked by chronic systemic inflammation (49, 50). A chronic inflammatory environment has been consistently implicated in the pathogenesis of sarcopenia by impairing protein turnover and synthesis and increasing skeletal muscle catabolism (51). Of particular relevance to SO, the increased adipose tissue provides an additional source of inflammation (52) that directly induces skeletal muscle atrophy (53, 54). Additionally, monocyte chemoattractant protein (MCP-1), a key chemokine in inflammatory cell trafficking, has been shown to be high in cardiac cachexia leading to increased monocyte and macrophage trafficking to adipose tissue resulting in an exacerbation of inflammatory cytokine secretion (55, 56).

### Oxidative Stress

Circulating markers of oxidative stress are increased in the setting of HFpEF and SO (57). The role of oxidative stress in HFpEF related obesity is not yet fully understood however, in individuals with SO, increased oxidative stress has been shown to be correlated with cardiovascular disease risk (57). Increased oxidative stress contributes to endothelial dysfunction by reducing the production and bioavailability of nitric oxide (NO) resulting in microvascular dysfunction which is a hallmark of HFpEF (58, 59). Impaired endothelial function can hamper blood flow and oxygen diffusion to the working muscle, thus potentially playing a role in exercise intolerance and the development of sarcopenia (25, 46). The sources of oxidative stress in HFpEF are multifaceted and have not yet been fully elucidated. Certainly, inflammation is characteristic of this HFpEF that is augmented by SO is a likely major contributor to increased levels of oxidative stress (60, 61). Cardiac ischemia and tissue hypoxia have also been implicated to play a role in HFpEF related oxidative stress (61, 62). In this respect, hypoxia related increases in uric acid have been reported in these patients (62). Therefore, xanthine oxidase, the ROS generating enzyme the catalyzes the oxidation of xanthine to uric acid could be a potential source of oxidative stress in HFpEF. Mitochondrial dysfunction that is consistently reported in HFpEF is an additional source of oxidative stress (63). Uncoupled endothelial nitric oxide synthesis (eNOS) due to reduced levels of the NO precursor L-Arginine and increased levels of the L-Arginine competitor asymmetric dimethylarginine is also a likely source of oxidative stress in these patients (62). Whether an activated renin angiotensin aldosterone system (RAAS) leading to increased levels of Angiotensin II is a source of oxidative stress in these patients remains controversial (64, 65). The discouraging lack of efficacy of RAAS targeted therapies in this syndrome supports some evidence that RAAS is not a major contributor to oxidative stress (65). As broad-level antioxidant therapies have generally not been successful at improving cardiovascular end-points (66), it is important that future research in HFpEF identifies the specific sources of excessive reactive species generation in order to develop targeted strategies to reduce oxidative stress.

### Endocrine Abnormalities

Multihormone hormone deficiency (MHDS) has been cited as a cause and consequence of HF (67, 68). Although relatively unexplored in the HFpEF cohort of HF, many of these patients present with one or more components of MHDS, these being an aberrant insulin like growth factor/growth hormone (IGF-1/GH) axis, abnormal levels of thyroid hormones, and androgens and insulin resistance (67–69). These hormone abnormalities are implicated in skeletal muscle catabolism and anabolic deficiency with downstream consequences on functional capacity (68).

Chronic low grade inflammation in obesity that may contribute to muscle wasting is regulated in part by adiponectin, leptin, and insulin controlling various inflammatory and anti-inflammatory processes (70–74). In obesity, homeostatic model assessment of insulin resistance (HOMA-IR) values are commonly increased (70, 71) and leptin is elevated, too (74). Adiponectin has been shown to be low and negatively correlated with BMI, plasma insulin, tumor necrosis factor alpha (TNF-α), and interleukin 6 (IL-6) (70). Heart failure with reduced ejection fraction (HFrEF) is known to have an uncommon adiponectin cascade compared to what is seen in obesity related conditions though it is unknown whether this also occurs in HFpEF due to limited research. One study that sought to evaluate this cascade in the two HF subtypes determined that in HFpEF and HFrEF leptin levels were high compared to matched healthy controls (72). Adiponectin was the same across groups until adjusted for age and BMI where it was higher in HF than controls (72). Adiponectin resistance has been determined to be present in HFpEF through higher adiponectin mRNA expression despite similar adiponectin secretion compared to healthy controls (73). Similar to obesity, adiponectin has a negative correlation to BMI in HF whilst leptin and insulin correlated positively with BMI (72).

### Mitochondrial Dysfunction

Skeletal muscle mitochondrial dysfunction is sentinel to the pathogenesis of sarcopenia through its dysregulation of myocyte function and viability (75). Impairments in mitochondrial function occur in parallel with decrements in strength throughout the spectrum of sarcopenia (76). In aging models of sarcopenia in mice, skeletal muscle mitochondrial dysfunction appears to be precursor to the development of sarcopenia as abnormalities are observed prior to declines in strength and muscle dystrophy (76). Potential contributors to HFpEF related mitochondrial dysfunction are thought to be chronic adrenergic activity, inflammation and oxidative stress (25, 77–81).

Additionally, skeletal muscle mitochondrial dysfunction results in a disruption in metabolism due to a reduced capacity for lipid oxidation. In the presence of increased circulating free fatty acids and triglycerides, incomplete fatty acid oxidation, and insulin resistance, an impairment in mitochondrial oxidative capacity could result in substrate overload and the previously described accumulation of lipid intermediates in the skeletal muscle (82). As previously mentioned, this accumulation of intramuscular fat is associated with SO and is positively associated with functional capacity in HFpEF (24).

### Iron Deficiency

Iron deficiency (ID) is highly prevalent in HFpEF and is more prominent in patients with more severe diastolic dysfunction. HFpEF related ID is thought to be a result of inflammation mediated reductions hepcidin, the principal regulator of iron absorption and tissue distribution (83–85). In addition, gastrointestinal irregularities that reduce gastric emptying and intestinal iron absorption are evident in these patients (83, 84, 86). In HFpEF patients, meta-analyses have shown ID to be correlated with reductions in exercise capacity, physical functional and worse QoL (83, 87). ID is hypothesized to play a role in lean mass abnormalities, sarcopenia and the consequential reductions in functional capacity via impaired cardiac and skeletal mitochondrial function as these organelles require iron for optimal oxidative phosphorylation enzyme function (83, 88).

### Disuse

Individuals with HFpEF are highly sedentary and therefore the resultant reduction in energy expenditure combined with disuse atrophy may contribute to SO in these patients (46, 89, 90). Sarcopenia is caused for the most part by physical inactivity. Patients with obesity that, however, remain physically active and maintain a preserved cardiorespiratory fitness, may not necessarily develop sarcopenia, possibly resulting in more favorable prognosis. Interestingly, a recent analysis of Korean individuals suggested that sarcopenia may also directly affect cardiac diastolic function, therefore impairing exercise capacity not only by affecting the peripheral determinants of cardiorespiratory fitness, but possibly by also affecting cardiac function (3).

**Figure 2:**
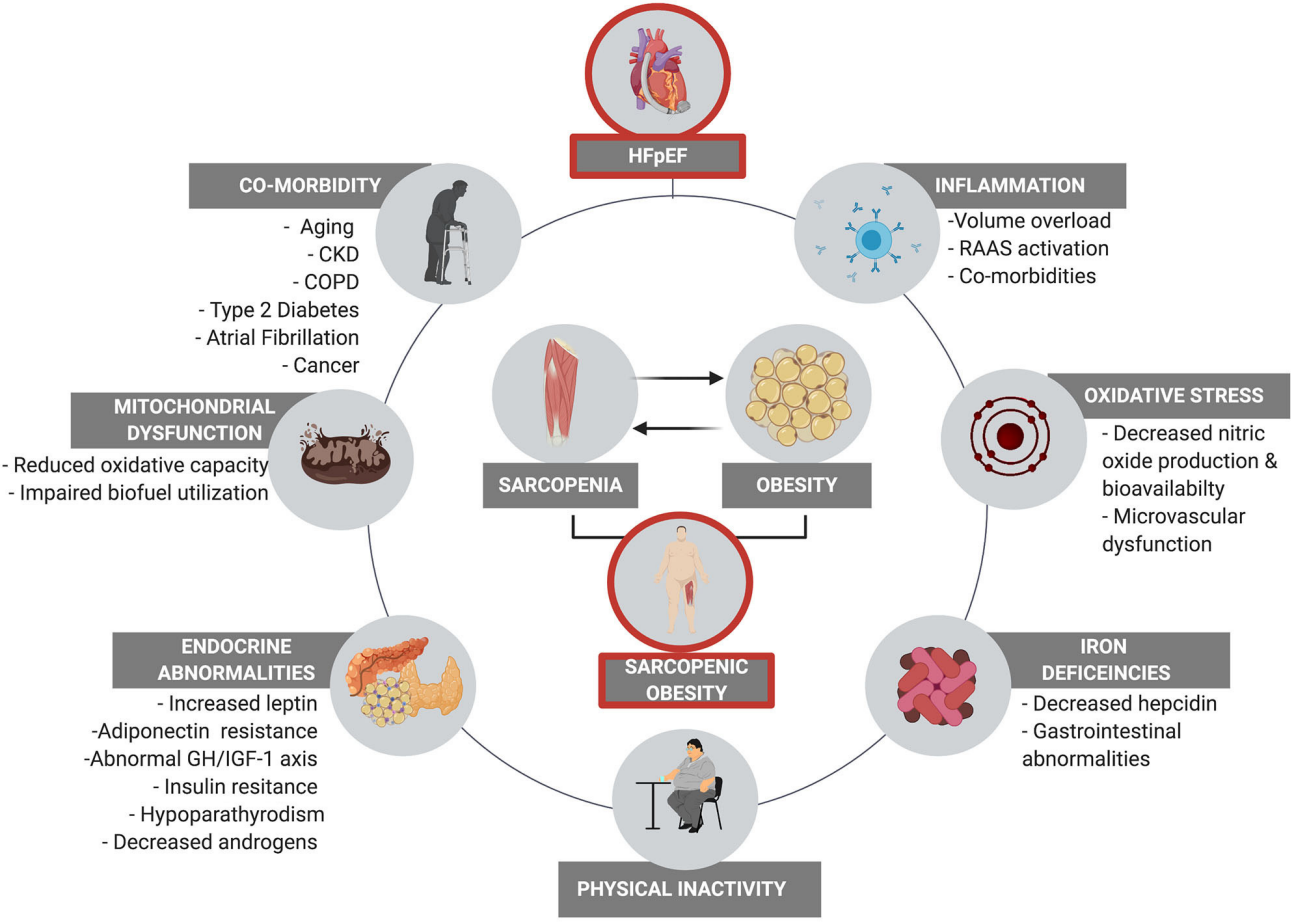
The downward spiral of SO and physical inactivity that culminates in reduced quality of life, increased hospitalization, and mortality. Interventions targeting SO in HFpEF are urgently warranted to reverse the associated detrimental downstream consequences.

Certainly, the co-morbidities and symptoms associated with HFpEF may contribute to exertional fatigue and the reduction in physical activity, thus augmenting a vicious cycle of disuse atrophy and increased adiposity (Figure 2). Although the role of physical inactivity in HFpEF related SO has not yet been clearly established, increasing physical activity levels through exercise training is one of the only therapies that has proven efficacious for improving exercise capacity (16, 91–93). The predominant mechanisms of these improvements in exercise tolerance are suggested to be mediated for the most part through beneficial skeletal muscle adaptations (15, 24, 91, 94).

## Current and Prospective Interventions for Sarcopenic Obesity in HFpEF

### Exercise Training

Despite relatively few randomized controlled trials that investigated exercise training in HFpEF, exercise training has consistently been shown to be efficacious at improving exercise capacity and QoL in this patient population (15, 91, 95–99). The implemented exercise interventions are predominantly aerobic in nature with exercise prescribed initially at a light-moderate intensity and gradually progressed to a moderate-vigorous intensity (15, 91, 95, 96, 98). High intensity interval training appears to be superior to moderate (100) continuous training at eliciting improvements in exercise capacity (91, 94, 97). As meta-analyses reveal limited changes in cardiac function following exercise training in HFpEF (15), the beneficial effects of training on exercise tolerance appear to be driven by peripheral improvements in skeletal muscle health and function (94). With reference to SO, there are currently no known trials in HFpEF that have investigated the effects of exercise on body composition as the primary outcome. In this respect, high intensity progressive resistance training is deemed the most effective mode of exercise to elicit an anabolic response and has shown positive results in other sarcopenic and cachexic populations (101–104). Such investigations are warranted in HFpEF patients with SO.

### Dietary Interventions and Bariatric Surgery

Effective nutrition interventions are urgently needed in the overall HF population and are especially lacking in HFpEF (105). To date, no nutrition interventions have specifically been performed in patients with comorbid HFpEF and SO. Healthy dietary patterns, specifically the Mediterranean diet (MedDiet) may be associated with a lower incidence of sarcopenia (106–108). Recently, a prospective study in Spain assessed adherence to the MedDiet in 991 patients diagnosed in the emergency department with acute HF on a primary endpoint of all-cause mortality. While the primary endpoint was not met, the secondary endpoint of rehospitalization was significant lower in those adhering to a MedDiet (HR = 0.76, 95% CI 0.62–0.93) (105, 109). The MedDiet is rich in fruits, vegetables, whole grains, legumes, and particularly unsaturated fatty acids (UFA), which have been associated with more favorable body composition, such as greater fat-free mass (FFM) and FFM to fat mass (FM) ratio as well as improved cardiac diastolic function and greater VO_2_peak in HFpEF patients (110). Of note, in the UFA-Preserved pilot study, a diet supplemented with foods rich in UFA such as extra-virgin olive oil, canola oil, and mixed tree nuts was associated with increased plasma levels of UFA in patients with obesity and HFpEF, showing that it is a feasible intervention in this population and associated with favorable changes in CRF (111). A larger ongoing randomized crossover trial, the UFA-Preserved 2 (NCT03966755), is designed to evaluate the effects of daily UFA supplementation through dietary sources on a primary outcome of change in dietary UFA and plasma UFA biomarkers, while also assessing changes in body composition, cardiac function and exercise capacity. Another healthy dietary pattern, the Dietary Approaches to Stop Hypertension (DASH) diet, is effective in treating hypertension and has also been shown to reduce clinic and 24-h systolic (155–138 and 130–123 mm Hg, respectively), and diastolic (79–72, and 67–62) blood pressure, arterial stiffness measured by carotid-femoral pulse wave velocity (12.4 to 11.0 m/s), improve diastolic left ventricular relaxation (difference in c=−4.2 ± 6.2) and chamber stiffness (difference in *k* = −81 ± 99 s-1) and ventricular-arterial coupling (difference in Ees:Ea= −0.2 ± 0.3) when combined with sodium restriction in HFpEF patients (80, 112). Recently, the GOURMET-HF trial assigned 66 patients with HFrEF and HFpEF discharged from the hospital after admission for HF exacerbation 1:1 to either a sodium restricted (<1,500 mg/day) DASH diet, which was home delivered, or usual care for 4 weeks. While the study did not meet the primary outcome of between group change in Kansas City Cardiomyopathy Questionnaire summary score, but participants assigned to the DASH diet demonstrated a favorable trend in rehospitalization at 30 days (11 vs. 27%) (113).

Protein supplementation, coupled with resistance exercise, may help increase appendicular muscle mass while reducing total and abdominal obesity as well as inflammatory biomarkers in women with sarcopenic obesity (114). While the most recent Heart Failure Society of America Scientific Statement recommended a protein intake of at least 1.1 g per kilogram per day (115), trials of protein and amino acid supplementation in patients with sarcopenia and HF have primarily performed in HFrEF patients and have yielded mixed results (116, 117).

Finally, the SECRET trial assigned 100 patients with obesity and HFpEF to a 2 × 2 factorial trial of exercise training, caloric restriction, both caloric restriction, and exercise training or control. The main effect of caloric restriction was weight loss (−7 kg, 95% CI −5−9) which resulted in an increase in VO_2_peak (+1.3, 95% CI 0.2–2.3), as well as a reduction in FM (−5 kg, 95% CI−4−6, *p* < 0.001) and percent body FM (−2%, 95% CI−1−3) (14). However, lean mass (−2 kg, 95% CI−1−3) was also reduced in these patients with caloric restriction alone, an important consideration in patients with SO (14). Notably, main effect of exercise revealed no loss of lean mass, suggesting that concomitant exercise training may help to preserve lean mass with caloric restriction (14). The currently enrolling SECRET-II trial (NCT02636439) is testing caloric restriction combined with aerobic exercise training alone vs. combined with resistance exercise training in patients with HFpEF.

In addition to weight loss induced by caloric restriction, there is a growing interest in understanding the role of bariatric surgery in this population. In HF, observational studies suggest clinical benefits of this intervention (11), however, randomized controlled trial are needed to confirm those findings. First, bariatric surgery induces significant weight loss to a greater degree than lifestyle intervention alone. This effect alone would allow to improve exercise capacity and quality of life (12, 13). The other potential effects of bariatric surgery are numerous, including the ability to reduce epicardial adipose tissue, which would be beneficial based on the above discussion. Moreover, bariatric surgery has been associated with reduced markers of systemic inflammation as well as localized adipose tissue inflammation (11), potential targets in patients with obesity and HFpEF.

Considering the lack of available therapies in HFpEF and prevalence of SO in this population, there is an urgent need to identify whether dietary pattern, protein supplementation or caloric restriction as well as bariatric surgery are able to improve outcomes in this population.

### Pharmaceutical and Nutraceutical

The discouraging lack of clinical outcomes pertaining to pharmacological therapies in HFpEF has provided a major challenge to cardiologists managing this syndrome. Despite a lack of beneficial outcomes pertaining to hospitalization and mortality in these patients, pharmaceutical therapies have resulted in clinical meaningful improvements in exercise capacity and QoL (96). In this respect, vasodilators, and chronotropic therapies appear to be efficacious, whist RAAS inhibitors have no effect (96). The DHART Trials investigating the effects of IL-1 blockade in HFpEF patients have shown promising improvements in exercise capacity and quality of life (77, 118, 119). In addition, inorganic and organic nitrates hold promise for improving exercise capacity in these patients (120–122). As with exercise training, there are no pharmacological strategies that have specifically targeted SO in human subject trials of HFpEF. As we continue to understand more about the pathophysiology of HFpEF related SO, future pharmacological or nutraceutical therapies targeting these aberrant molecular pathways in combination with exercise training may be a powerful approach to combat SO.

## Conclusions

In summary, SO is prevalent in patients with HFpEF and has noteworthy adverse consequences on end point outcomes, health related outcomes and patient reported QoL. Although the multifaceted pathophysiology of HFpEF related SO is not yet fully understood, inflammation, oxidative stress, hormonal imbalances, iron deficiency, and sedentary lifestyle appear to contribute to this condition. In addition, the plethora of comorbidities that accompany this syndrome further exacerbate the physiological milieu implicated in SO. Moreover, being obesity a major risk factors for several comorbidities, including coronary heart disease, future research should try to assess the diverse effects of obesity and HFpEF on clinical outcomes.

Whilst exercise training as well as some dietary and pharmaceutical approaches appear to be efficacious at improving exercise capacity and QoL, there is a lack of interventional trials that specifically target SO in these patients. There is therefore an urgent need for lifestyle, pharmaceutical or nutraceutical approaches to address SO toward improving clinical and disease modifying outcomes in HFpEF.

## Author Contributions

DK, NB, HB, and SC contributed to the content of the manuscript. DK, NB, HB, and SC edited manuscript drafts. DK, NB, HB, and SC approved the final version of the manuscript. All authors agree to be accountable for the content of the manuscript.

## Conflict of Interest

The authors declare that the research was conducted in the absence of any commercial or financial relationships that could be construed as a potential conflict of interest.
